# Characterization of Glass Powder from Glass Recycling Process Waste and Preliminary Testing

**DOI:** 10.3390/ma14112971

**Published:** 2021-05-31

**Authors:** Ester Gimenez-Carbo, Lourdes Soriano, Marta Roig-Flores, Pedro Serna

**Affiliations:** 1 Institute of Concrete Science and Technology (ICITECH), Universitat Politècnica de València, 46022 València, Spain; lousomar@upvnet.upv.es (L.S.); pserna@cst.upv.es (P.S.); 2 Department of Mechanical and Engineering Construction, Universitat Jaume I, 12071 Castellón de la Plana, Spain; roigma@uji.es

**Keywords:** glass, pozzolane, concrete, mortar, fine cullet

## Abstract

This work studies the possibility of incorporating different proportions of glass powder from the waste glass (rejected material called fine cullet) produced during the glass recycling process into the manufacturing of mortar and concrete. For this purpose, the material is characterized by its chemical composition and pozzolanic activity, and the shape and size of its particles are studied. It is then incorporated as a substitute for cement into the manufacturing of mortar and concrete at 25% and 40% of cement weight, and its effect on setting times, consistency, and mechanical strength is analyzed. Its behavior as a slow pozzolan is verified, and the possibility of incorporating it into concrete is ratified by reducing its cement content and making it a more sustainable material.

## 1. Introduction

Concrete is the most widespread construction material in the world. In 2017, around 45 million tons were used in Spain [1] and its consumption worldwide was estimated at 10 billion tons [2]. With these data, it can be stated that concrete consumption equates to approximately 1.7 tons/year per person [3]. The main concrete component is cement, and the chemical and thermal combustion processes involved in its production are a major source of carbon dioxide (CO_2_) emissions. Each ton of cement emits around one ton of CO_2_ into the atmosphere from both the burning of fuel and the decarbonation of the raw materials of cement [2]. As more than 4 billion tons of cement are produced annually, it can be stated that it is responsible for about 8% of global CO_2_ emissions [4].

For this reason, the use of solid waste material or industrial byproducts as a partial cement replacement (supplementary cementitious material) in concrete is a viable strategy for reducing the use of Portland cement and for, therefore, reducing the environmental and energy impacts of concrete production [5,6].

In the line of work of the study of incorporating waste into the concrete matrix, the feasibility of such incorporation of waste from the glass recycling industry has been investigated for several years as part of either mortar, concrete and cement [7,8,9,10,11], or geopolymers [12,13,14], although the latter use is beyond the scope of our study. This is due to its chemical composition with high silica content in an amorphous state. This led us to think that, with the right size particles, waste obtained from glass can behave like pozzolanic material. The effect of glass powder particle size has been studied and it has been concluded that the particle size directly influences the pozzolanic behavior; the finer the glass powder is, the higher its pozzolanic reactivity is [15]. However, the alkali–silica reaction is usually associated with larger particles with amorphous silica in their composition with minimum sizes ranging from 150 µm to more than 1 mm, depending on the composition of the glass and the composition of the concrete [16].

The incorporation of this material into concrete will lead to improvements in two different areas. A waste product will be recovered directly from either inert solid waste management plants (those not deposited in glass recycling containers) or the rejection of material that cannot be used as cullet for producing recycled glass. This will reduce not only the volume of waste that has to be disposed at landfill sites, but also the amount of cement or fine aggregate in concrete, thus reducing the carbon footprint of the most widely used building material in the world. This would, therefore, contribute to the achievement of SDG12 “Responsible Consumption and Production” and SDG13 “Climate Action”.

In 2019, 958.939 tons of glass were collected in Spain, of which 56,628 tons came from urban solid waste plants and the rest from specific glass containers, with a recycling rate of between 72% and 77% [17]. Most of the glass collected for recycling is crushed to obtain the cullet, which will be the raw material for recycled glass. During this process however, some material is discarded because it is not the right size or given impurities in waste. This material is called fine cullet [18] and it can be ground to particle size, which makes it suitable for use in concrete. It can be added as a partial or total substitute for fine aggregate or as a partial substitute for cement because, given its amorphous structure and high amorphous silicon and calcium contents, glass is theoretically pozzolanic or even cementitious in nature when finely ground [19]. This use of glass as a partial substitute for cement in concrete confers the material more value and makes it a sustainable and environmentally friendly material.

At the end of the 1990s, several research works began to study the possibility of incorporating glass powder (GP) into the concrete composition as a substitute for part of the fine aggregate [6,19], as a substitute for cement [15,20], or as simply a part of it [21]. These studies aimed to verify how the incorporation of this recycled material would affect the properties of concrete, and different experiments on mortars commenced.

Rashad [19] published an overview of the studies carried out until 2014. The conclusions established that contradictory results were often obtained about the effects of incorporating GP as a sand substitute. Some conclusions were reached, such as the incorporation of glass led to higher temperatures during hydration, increased the setting time, and bleeding and segregation augmented with higher glass content, while density decreased with rising glass content in mixtures. No conclusive conclusions were reached for other properties [22]. In some studies, including more glass enhanced workability [23,24], but it decreased in others [9,25]. Mechanical strength increased in some studies, but not in others [9,22,26], depending on the age of the test and the replacement rate of the glass powder. These variations in the results are due to the different shapes and sizes of glass particles, the proportion of cement in the concrete and their substitution percentages.

The pozzolanic effect that would justify the partial cement substitution for GP is directly related to the size of the ground glass particles, which is bigger the finer the GP [15]. A rising curing temperature also increases pozzolanic activity, and its incorporation into the matrix of mortars diminishes their expansion [15].

The use of GP in cement raw mixes during clinker burning [21] does not offer good results as it has an adverse effect on stable furnace operation and lowers the C_3_S content in the clinker. Nonetheless, as it does not lead to new minerals forming in the clinker, it could be added in small fractions.

All studies clearly reveal that this topic is complex and further research is needed before introducing GP into the concrete matrix becomes common practice. However, the obtained results have shown that GP can contribute to achieve the desired mechanical strengths. 

In Spain, part of the glass recovered during the recycling process is reused for the purpose of producing new glass containers. In this process, part of that material cannot be reused due its inadequate size and has to be sent as waste material to solid inert waste landfills. One of the novelties of this research is that we used this waste material (fine cullet) to perform this research. Other researchers also used this same concept [27] in order to produce concrete but without replacing part of the cement content with it.

Therefore, the objectives of this work are to characterize GP obtained from the glass recycling waste produced in Spain, and to incorporate this GP into mortar and concrete in different proportions to study its influence on their properties in fresh and hardened states.

## 2. Materials and Methods

The experimental study was divided into three sections. In the first section, the physico-chemical characteristics of GP were studied to measure its pozzolanic potential. Second, different cement mortars were manufactured in which one part of the cement was replaced with GP at 0%, 10%, 25% and 40%. Its consistency, setting time, and mechanical strength were then analyzed. Finally, different concrete mixes were manufactured, in which one part of the cement was replaced with GP at 0%, 25% and 40%. The consistency and evolution of the compressive strength of these concretes were studied.

### 2.1. Materials

#### 2.1.1. Glass Powder

Glass powder was obtained from a product donated by the company FCC ámbito (Sagunto, Spain). This company has a plant that receives and processes glass, where the material obtained during selective collection arrives. From this material, a cullet is obtained and sent as raw material to the main glass manufacturers. It is reintroduced into the market which, thus, favors fulfilling circular economy principles. During the process of recovering the product obtained from glass recycling, a waste glass (fine cullet) is produced destined for landfills. This is the material that was subsequently ground to obtain the GP used in this work. Grinding was carried out in an industrial pendular mill, which has a set of pendulums with rollers at the ends that grind material at high pressure against a grinding ring. Pressure is created from the high-speed rotation of the star from which the pendulums hang.

#### 2.1.2. Other Components

The cement used to prepare paste, mortar, and concrete was CEM I 42.5R cement, which was supplied by the CEMEX factory in Buñol (Valencia, Spain).

The sand used for mortars was a mixture of three fractions to obtain a similar size distribution to that of normalized sand. The sand was siliceous and came from SILICAM (Liria, Valencia, Spain) and had a fineness modulus of 4.3. For the concrete mixes, three gravels of sizes 4/8, 8/16 and 16/20 were used as coarse aggregates, while a combination of two sand types was used as fine aggregates by combining a coarser sand 0/6 named “red sand” and a finer sand 0/2 called “white sand”. All these aggregates are silico-calcareous aggregates. For concrete mixes, Sika Viscocrete 5970 (polycarboxylate-based) was used as a superplasticizer (Sika, Madrid, Spain). The amount of additive used was 0.5% by weight of binder.

### 2.2. GP Characterization Methods

Several techniques were followed to characterize GP. The chemical composition of GP was measured with a Philips Magix Pro spectrometer (Philips, Amsterdam, The Netherlands) via the X-ray flourescence (XRF) technique. The equipment used to measure granulometric distribution was a Mastersizer 2000 (Malvern Instruments, Malvern, UK). The technique that employed the equipment was laser diffraction. GP was dispersed in water. Bruker AXS D8 Advance equipment (Bruker, Billerica, Germany) was employed to measure the X-ray diffraction (XRD) analyses. The range of measures went from 10 to 70 2θ degrees, with Cu Kα radiation at 20 mA and 40 kV, and a 2 s accumulation time in a 0.02 angle step. Finally, the GP morphology was observed under a field emission electronic microscope. The employed equipment was an FESEM ULTRA 55 scanning electron microscope. (Zeiss, Jena, Germany) Samples were carbon-coated in order to observe them and images were taken at 2 kV.

For the thermogravimetric (TG) analyses, plain cement paste and cement/GP pastes were manufactured. The pastes were made under the same conditions as the mortars but without aggregate. The mixture was put inside a cylindrical plastic container with a hermetic closing until the testing age. For a given curing age, a paste sample was taken and milled in an agate mortar with acetone to stop the hydrating process. This ground sample was filtered and dried off for 30 min at 60 °C. Finally, it was sieved through a 125 μm sieve and was ready for further TG analyses. For field emission scanning microscopy (FESEM) purposes, a piece of paste was placed in acetone for 1 h and dried off for 30 min at 60 °C. 

The TG analyses were carried out using a TGA 850 Mettler-Toledo module (Mettler-Toledo GmbH, Giessen, Germany) to take measurements up to 1100 °C. The utilized equipment had a horizontal electrobalance, a furnace, and temperature sensors connected to a computer in which data were recorded and processed by specific equipment software. The ultramicrobalance resolution was 0.1 µg. For the analysis of the cement pastes with GP, 100 μL aluminum crucibles were used with sealed covers, which had microholes to provide a self-generated water vapor atmosphere, which, in turn, allowed better resolution of the TG curves. For pastes, the selected temperature range was 35–600 °C with a 10 °C/min heating rate and a nitrogen atmosphere at a 75 mL/min flow rate.

The Frattini test is a test for determining pozzolanic activity. This test is described in Standard UNE-EN 196-5 [28]: “Methods of testing cement. Part 5: Pozzolanicity test for pozzolanic cement”. By this standard, pozzolanicity is assessed by comparing the concentration of calcium ions, expressed as calcium oxide, present in the aqueous solution that comes into contact with the hydrated cement after a fixed period of time, with the quantity of calcium ions capable of saturating a solution with the same alkalinity. Cement is considered to meet the test, i.e., give a positive result, if the concentration of calcium ions in the solution is lower than the saturation concentration. To test the pozzolanity of GP, three mixtures were manufactured: 100% Portland cement, 25% replacement of Portland cement with GP and 40% replacement of Portland cement with GP.

### 2.3. Tests on Mortars and Concrete

#### 2.3.1. Mortars

The cement mortar was prepared according to the specifications of the Spanish standard UNE-EN 196-1 [29], where the mixing method and mechanical strength test are described. The water/binder ratio was 0.5, with the binder as the Portland cement or Portland cement plus GP in the cement replacement series. The mortar mixtures are summarized in Table 1.

The consistency of mortar was determined using the flow table method UNE-EN 1015-3 [30]. The flow value is determined by measuring the mean diameter of a test sample. The diameter of the spread mortar is measured in two directions at right angles to one another using calipers. Both results are reported.

For the setting time test according to UNE EN 196-3 [31], the standard consistency of cement corresponds to 28% water content in cement weight. For a kneading of 500 g of cement, the water content corresponds to 140 g. This same amount of water was maintained in the pastes in which 25% and 40% of cement were replaced with GP.

The mechanical strengths of the 40 mm × 40 mm × 160 mm prismatic mortar specimens were determined according to UNE EN 196-1 [29]. Samples were stored in a mold in a humid atmosphere for 24 h. After demolding, they were stored underwater until the strength test. At the required age, the specimens were taken from their wet storage, broken in flexure, and each half was tested for strength in compression.

#### 2.3.2. Concrete

Concrete was made in an electric mixer (40 L capacity) in which batches of 20 L and 25 L were prepared. Table 2 shows the composition of the concrete mixes with the 25% and 40% replacement rates of cement with GP.

In order to check the effect on the fresh concrete structure of GP incorporation into the concrete matrix, slump and compressive strength tests were carried out on concrete mixes. 

The slump test was used to measure the consistency or workability of the fresh concrete following UNE-EN 12350-7 [32] and for testing the compressive strength of the concrete, the UNE-EN 12390-3 [33] standard was used, and 150 mm cubic specimens were cast. Specimens were loaded to failure in a compression testing machine in compliance with EN 12390-4 [34]. The maximum load sustained by specimens was recorded and the concrete compressive strength was calculated.

## 3. Results and Discussion

### 3.1. Physico-Chemical Characterization

The chemical composition of the GP is provided in Table 3. As we can see, the major oxide components were SiO2, Na2O, and CaO. The sum of these three oxides exceeded 90% total composition.

**Figure 1:**
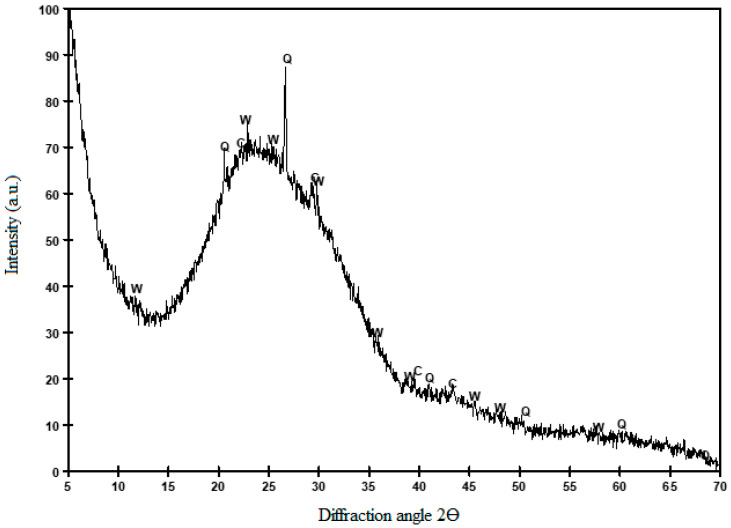
X-ray diffractogram of the studied GP.

The obtained X-ray diffractograms are represented in Figure 1, which reveals that the studied GP is a material with a large amorphous phase, as indicated in the diffractogram by a wide deviation from the baseline within the 10–35 2θ range. Some crystalline compounds were also detected: quartz, SiO_2_ (Q) (PDFcard 331161), and traces of calcite, CaCO_3_ (C) (PDFcard 050586) and wollastonite, CaSiO_3_ (W) (PDFcard 100489).

**Figure 2:**
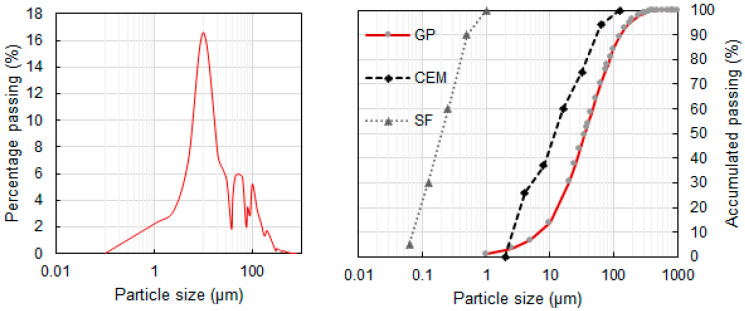
Particle size distribution of GP (**left**) and a comparison with CEM and SF of this study (**right**).

Previous research has indicated that GP size is critical for the material to show pozzolanic activity [35]. The size distribution of the GP was evaluated 3 times. Figure 2, left, displays the curves obtained for the three measurements taken of the GP, where we can see that the size of most particles was around 10 microns. Figure 2, right, shows the accumulated passing size distribution of these three GP measurements compared to not only the cement used in this study but also to a silica fume. The graph shows that the obtained GP fraction was slightly coarser than CEM. SF had, on the other hand, a much smaller particle size, with most of its particles being less than 1 μ. SF has been chosen as the reference pozzolan because it is widely used and appears in the Spanish standard. The milled GP had a mean diameter of 25.6 µm with 90% of particles below 51.6 µm and only 10% of particles below 4.4 µm.

To date, it has not been possible to obtain smaller GP sizes following an industrial grinding procedure. In future studies, finer GPs should be studied to verify the influence of their particle size distribution on their pozzolanic character and to estimate if it is necessary to obtain alternative grinding methods to obtain a finer product.

**Figure 3:**
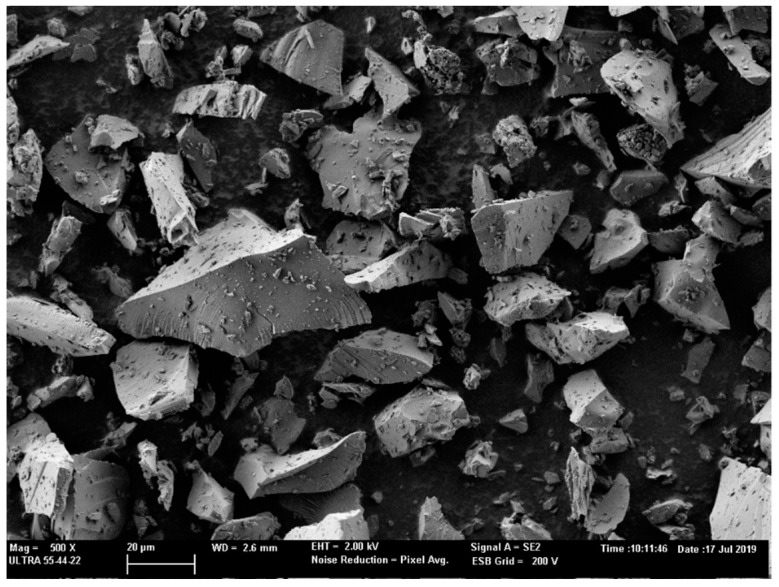
Shape and size of the GP particles seen by FESEM at 500× magnification.

The morphology of GP particles was evaluated by FESEM. As Figure 3 depicts, GP had several particle sizes. Particles had smooth spit-shaped surfaces.

#### 3.1.1. Pozzolanic Activity

Two methods were chosen to analyze the material’s pozzolanic reactivity, which are methods with different methodologies that do not always lead to the same result. Fratini is the normative method [28] and TG, which has already been used to analyze the pozzolanic behavior of other waste material types [36,37].

##### Pozzolanic Reactivity (Fratini Test)

**Figure 4:**
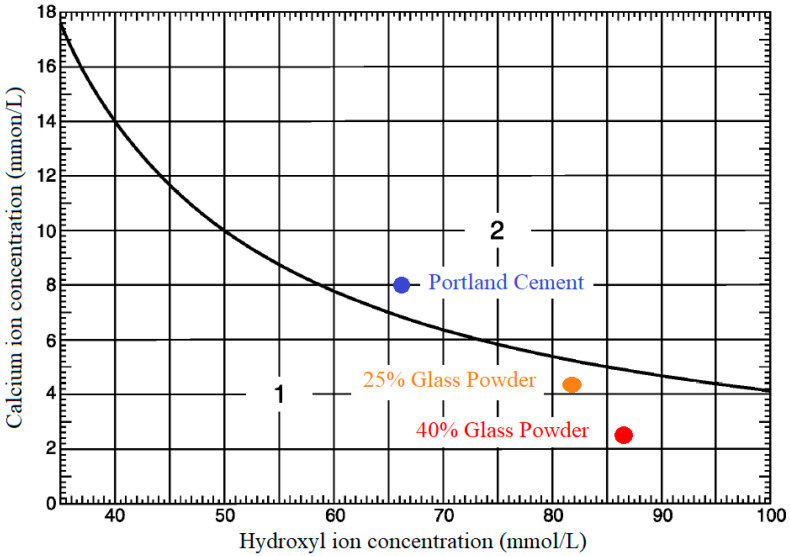
Calcium ion concentration vs. hydroxyl ion concentration in GP and CEM.

In order to test the pozzolanity [28] of GP, several solutions were manufactured that contained Portland cement, 25% replacement of Portland cement with GP, and 40% replacement of Portland cement with GP. After preparing solutions and keeping them in thermostatic compartments for 15 days, the following results were obtained (Table 4):

Once the concentrations of hydroxyl and calcium ions, expressed as calcium oxide, in the solution had been obtained, they were featured in Figure 4, which shows the saturation concentration of calcium ions, expressed as calcium oxide, according to the concentration of hydroxyl ion at 40 °C). Their pozzolanic character was determined according to the position they occupied (area 1 or area 2).

Figure 4 shows the results obtained with the Portland cement along with the 25% and 40% GP substitutions. As expected, the samples with the different GP percentages displayed the pozzolanic character.

##### Thermogravimetric Analysis

The reactivity of GP on the fixation of portlandite generated during the hydration of the Portland cement was studied by means of TG analysis of the corresponding cement/GP pastes at curing ages of 7, 28 and 210 days. The chosen percentage of cement replacement with GP was 25%. 

In the cement pastes with replacement materials, fixed lime was a function of the calcium hydroxide contained in the control paste (only cement as a binder). The following expression (1) was used to calculate the fixed lime percentage [37]:

(1) $$\%\;\mathrm{Hydrated}\;\mathrm{fixed}\;\mathrm{lime}=\frac{\left|{\mathrm{CH}}_{C}\cdot C_{\%}\right|-{\mathrm{CH}}_{P}}{\left|{\mathrm{CH}}_{C}\cdot C_{\%}\right|}\cdot 100$$

where CH_C_ is the quantity of portlandite (lime) present in the control paste, CH_P_ is the quantity of portlandite present in the paste with GP and C_%_ is the percentage of cement present in the paste (0.75 in this case).

**Figure 5:**
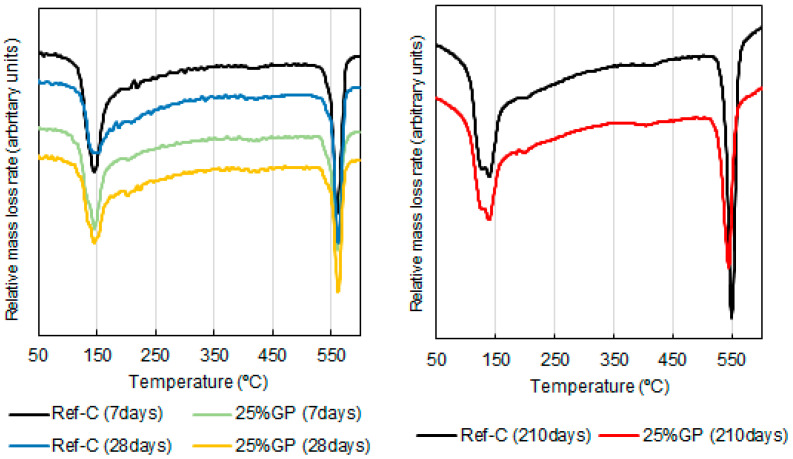
DTG curves of a reference paste and a paste with 25% GP at aging days 7 and 28 (**left**) and 210 (**right**).

The fixation analyses with the 25% cement replacement with GP showed negative lime fixations for both the 7 and 28 curing day periods. At 210 curing days, the fixed lime value was 3.4. In this case, at 7 curing days, the paste with the waste glass had a negative value of −0.61%, which increased to −16.21% at 28 curing days. Negative lime fixation percentages are typical of long-term reactive pozzolans, such as fly ash [36]. The negative fixed lime values were due to the filler or particle effect, which made the cement hydrate more quickly and, therefore, produce more portlandite than the amount corresponding to this curing age. DTG curves (Figure 5) have been shown to display the hydration products that had formed.

Payá et al. [37] described the characteristic areas of mass loss in the DTG curves. At around 100–180 °C the water mass loss derived from the calcium silicate hydrates (CSH) and ettringite (ET) occurred. At 180–240 °C, the dehydration of calcium and aluminosilicate hydrates (CAH and CASH) occurred, and, finally, the dehydroxilation of portlandite occurred around 520–580 °C. In the control paste and 25% GP paste, the formed hydration products were mainly CSH and the portlandite. The presence of CAH and CASH were hardly perceptible in these pastes. Although this doesn’t mean that these products didn´t form, they weren’t the majority.

#### 3.1.2. Scanning Electronic Microscopy (SEM)

**Figure 6:**
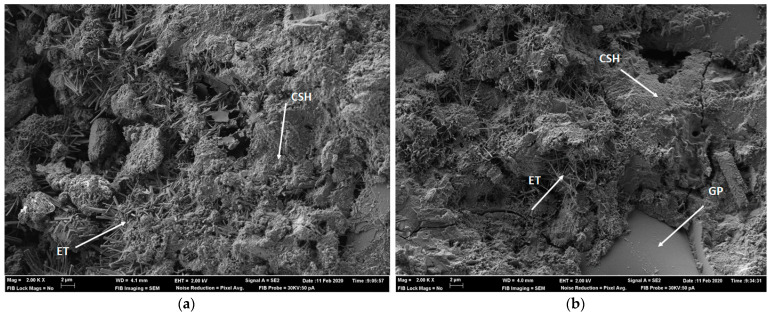
SEM analysis images for the control paste (**a**) and the GP paste (**b**) at 2000× magnification.

The micrographs obtained via FESEM of pastes at 28 curing days are presented in Figure 6. Figure 6a presents the control paste. The main observed reaction products are calcium silicate hydrate (CSH) and ettringite (ET). Figure 6b shows the GP paste, where the reaction products are the same as the control paste. In addition, a GP particle can be seen to be apparently unreacted, but completely surrounded by reaction products.

### 3.2. Test on Cement Mortars

#### 3.2.1. Consistency

When replacing cement from the mortars with GP, consistency only slightly reduced for the 40% contents. Those mixes with the 25% cement replacement obtained the same consistency results as the reference mortar mix. Table 5 shows the consistency and density results of the prepared mortars. Similar results have been found in research on glass powder mortars [38]. A slight reduction in density was observed in mortars with GP. This result is similar to those obtained by other researchers [39] and it is due to the lower specific gravity of GP.

#### 3.2.2. Setting Time

To carry out the setting time test, a standard consistency paste was made according to UNE-EN 196-3 [31], which corresponds to 28% water over cement weight. To know the differences of this property when substituting part of the cement with GP, pastes were manufactured with 25% and 40% cement substitutions, but with the same amount of water because it was necessary to obtain the standard consistency paste. Paste compositions are found in Table 6.

The obtained results show a delay in the final setting time in the pastes with GP, but with similar times at the start of the setting time.

#### 3.2.3. Mechanical Studies on Mortars

The cement mortar specimens made by replacing a percentage of cement with GP (Table 1) were tested at 7, 28, and 60 days to know their strength to flexion and compression, and to obtain their activity index. 

Three batches for each type of mortar (control, GP25, and GP40) were produced. From each batch, 3 specimens were tested to flexural strength at the ages of 7, 28 and 90 days (27 mortar specimens tested in total), and 6 specimens were tested to compression strength at the same ages (54 specimens tested in total).

**Figure 7:**
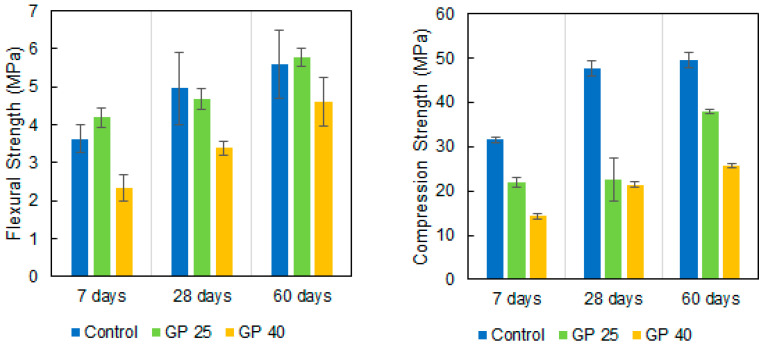
Flexural (**left**) and compression strength (**right**) values of the mortar mixes with different cement replacement rates with GP (average and standard deviation).

The obtained flexural strength values (Figure 7, left) had similar values for the control mortar and the mortar with 25% cement replacement with GP. However, flexural strength slightly reduced when the replacement rate was increased to 40%.

As previous research has shown [40], replacing a percentage of cement with glass powder reduces its mechanical strength. The pozzolanic effect of GP was seen when comparing compression strengths (Figure 7, right). With the variation in compressive strength from 7 to 60 days, the increase in the control mortar strength was around 36%, while the specimens with a partial cement replacement with GP obtained higher values of around 42%. This effect was more pronounced when analyzing the compressive strength values obtained at 28 and 60 days. In this case, the compressive strength value of the control mortar only increased by 3.6%, while the mortars with GP substitution by far exceeded this value with an increase of more than 40% for the 25% substitution. 

**Figure 8:**
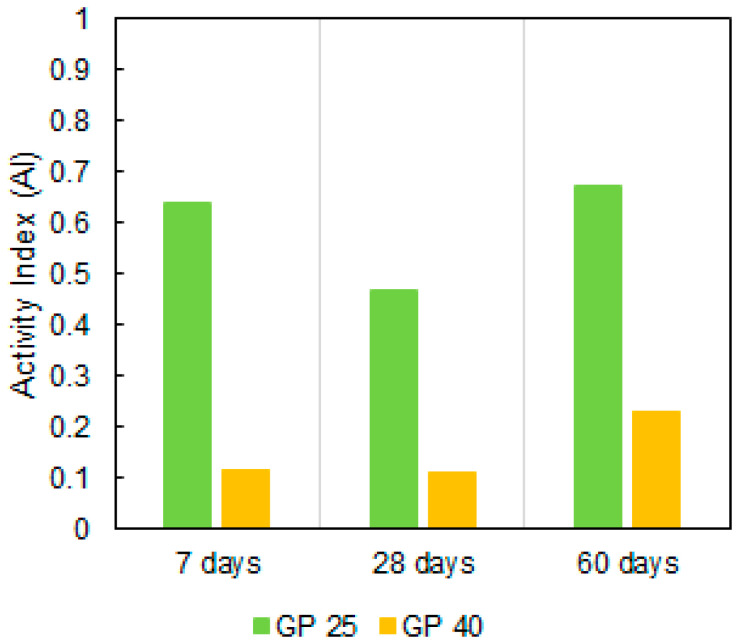
Activity Index (AI) of the analyzed mortar mixes.

Pu [41] proposed the following expression to calculate and analyze the activity of any cement replacement material:

(2) $$\mathrm{AI}=1-\frac{Compressive\;strength\;of\;control\;mix*\mathrm{GP}\;percentage}{Compressive\;strength\;of\;\mathrm{GP}\;mix*100}$$

where GP is the percentage of cement replacement with GP. The positive AI values indicate a reactivity of GP and the GP replacement had a positive effect on compressive strength. Although the GP40 value is positive, it is very low because it is not a very reactive pozzolan, so its pozzolanic effect does not compensate for the dilution effect of eliminating 40% cement. Figure 8 shows the Activity Index (AI) of the analysed mortars mixes.

### 3.3. Testing Concrete

Concrete was produced in two batches of 25 L per type of concrete (control, GP25 and GP40). From each batch, two specimens were produced to test compression strength at the ages of 7, 28, and 60 days (data could not be obtained at 60 days due to COVID-19 restrictions). The density measured for these mixes was: (1) control concrete 2375 kg/m^3^, (2) GP25 concrete 2220 kg/m^3^, and (3) GP40 concrete 2183 kg/m^3^.

#### 3.3.1. Consistency

**Figure 9:**
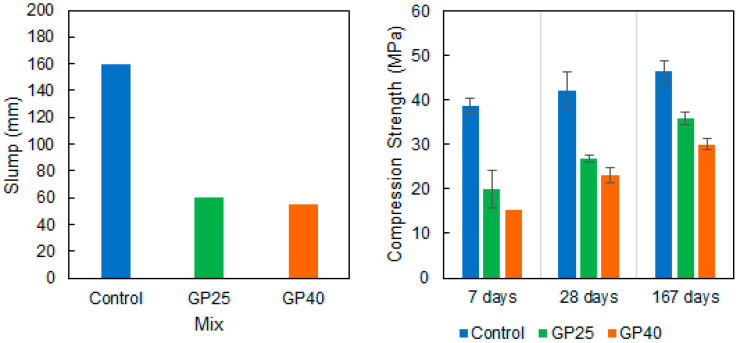
Slump (**left**) and compression strength (**right**) values of the concrete mixes with different replacement rates of cement with GP (averages are bars and standard deviations are whiskers).

Figure 9, left, shows the results of the slump tests performed with the three concrete mixes. The three mixes had the same water and superplasticizer contents and, hence, this variation occurred only due to the differences between cement and GP. The 25% and 40% replacements of cement with GP brought about a decrease of around 100 mm in the slump test, which is considerable. This decrease could have been produced by the larger specific surface area and the more angular texture of GP particles compared to the cement that was replaced. This decrease may potentially be compensated for by increasing the superplasticizer content.

#### 3.3.2. Compressive Strength

The concrete specimens made by replacing a percentage of cement with GP (Table 2) were tested at 7, 28, and 167 days (due to the COVID-19 crisis, it was impossible to access the lab to obtain data at 60 days as originally planned) to know their compression strength (Figure 9).

The results show the pozzolanic behavior of the concretes into which GP was incorporated. If we look at compressive strength evolution, we find that the control mortar showed a 7.93% increase in resistance at 28 days, and one of 16.46% at 167 days. These values were higher for the 25% substitution of cement for GP with 25.96% and 44.67% at 28 and 167 days, respectively. These values were even higher for the concrete with the 40% substitution of cement for GP in which increased resistance would be 34.65% and 49.59% at 28 and 167 days, respectively.

This indicates that despite the slight decreased strength in those concretes in which part of cement was substituted for GP, pozzolanic behavior was observed along with greater strength gain over time. These results are similar to those obtained by Tamanna and Tuladhar [39] with different percentages of cement substitution.

## 4. Conclusions

This research studied the use of glass powder from the waste generated in the glass recycling process (fine cullet) as a partial cement replacement in mortars and concrete. The experimental results obtained in this work allowed us to conclude that:

GP acts as very slow pozzolan. Mortars and concretes with GP cement substitution always showed a low compressive strength compared to the controls. The decrease in strength was greater the higher the percentage of GP substitution. However, when analyzing the activity index, it was observed that this decrease in strength decreased over time, which confirms GP’s performance as a slow pozzolan. This behavior was also verified by the results obtained in the pozzolanic reactivity test (Fratini) and in the thermogravimetric analysis. Replacement percentages of 40% GP may be excessive, but replacements of up to 25% can guarantee enough strength for elements that are not extremely demanding. 

The incorporation of GP into mixtures did not strongly influence concrete setting time, but slightly delayed the final setting time. The density of mortars and concretes with GP decreased, and this decrease was greater the higher the percentage of substituted cement due to the low specific gravity of glass powder.

Finally, GP lowered the Abrams cone value due to its larger specific surface area and the more angular texture of GP particles compared to the cement that was replaced.

All the findings of this experimental study indicate that using GP from glass recycling process waste in mortar and concrete production is possible. This is a very important fact because it proposes replacing a portion of cement with a material that is normally taken to landfills. The outcome of this research aims to contribute toward sustainable development by reducing the consumption of cement as well as a reducing the amount of glass waste going to landfills. Future research will study lower replacement percentages to find the optimum ones in terms of ecological benefits and minimum performance loss.

## Figures and Tables

**Table 1:** Mortar mixtures replacing cement with GP.

Type of Mortar	Cement (g)	Glass Powder (g)	Sand (g)	Water (g)
Control	450	0	1350	225
Cement Replacement				
GP25	337.50	112.50	1350	225
GP40	180	270	1350	225

**Table 2:** Concrete mixtures replacing 25% or 40% cement with GP (kg/m^3^).

Concrete Mixtures	Cement ^1^	GP	Total Water	White Sand	Red Sand	Gravel 4/8	Gravel 8/16	Gravel 16/20	Superplasticizer
Control	280	0	190	211	897	146	402	183	1.4
25%GP	210	70	190	211	897	146	402	183	1.4
40%GP	168	112	190	211	897	146	402	183	1.4

^1^ CEM I 42.5R.

**Table 3:** Chemical composition of the studied GP (wt.%).

Na_2_O	MgO	Al_2_O_3_	SiO_2_	P_2_O_5_	SO_3_	K2O	CaO	Other	LOI ^1^
10.13	1.17	2.51	69.43	0.15	0.84	1.15	11.93	1.38	1.31

^1^ LOI loss of ignition determined at 950 °C during 1 h.

**Table 4:** The pozzolanic reactivity test results.

Type of Concentration	Portland Cement	25% Glass Powder	40% Glass Powder
[OH] (mmol/L)	65.45	82.01	86.05
[CaO] (mmol/L)	8.05	4.31	2.75

**Table 5:** Flow table results and density of the mortar mixes with cement replacement with GP.

Type of Mortar	Water Content (g)	Slump Flow (mm)	Density (kg/m^3^)
Control	225	171.3 ± 0.7	2273 ± 6
GP25	225	185.0 ± 0.5	2199 ± 12
GP40	225	135.5 ± 0.6	2156 ± 2

**Table 6:** Paste compositions and setting times.

Paste of Standard Consistency	Composition				Setting Time		
	Cement (g)	GP (g)	Water (g)	a/b	Initial (h)	Distance (mm)	Final (h)
Control	500	0	140	0.28	3:10	5	4:40
GP-25	375	125	140	0.28	3:10	7	5:00
GP-40	300	200	140	0.28	3:00	7	6:20

## Data Availability

Data is contained within the article.
